# THE ASSOCIATION BETWEEN PSYCHOTROPIC DRUGS AND PHYSICAL FUNCTION IN HOSPITALIZED OLDER ADULTS WITH DEMENTIA

**DOI:** 10.1093/geroni/igad104.2417

**Published:** 2023-12-21

**Authors:** Nayeon Kim, Barbara Resnick

**Affiliations:** University of Maryland Baltimore, Baltimore, Maryland, United States; University of Maryland, Baltimore, Maryland, United States

## Abstract

The aim of this study was to test if the use of psychotropic medications (antipsychotics, sedatives, antiseizure, antidepressants, and anxiolytics) was associated with physical function in hospitalized older adults living with dementia. This study was a secondary data analysis using baseline data from the Function Focused Care for Acute Care Using the Evidence Integration Triangle (FFC-AC-EIT) study. FFC-AC-EIT is an ongoing randomized controlled trial and this study included the first 290 participants. A path analysis was conducted to test the relationship between psychotropic drugs and physical function while controlling for age, gender, race, comorbidities, cognitive function, neuropsychiatric symptoms of dementia, and pain. Overall, 63.4% of participants took one or more psychotropic drugs, with the most used psychotropic drugs being antidepressants (39%), followed by antiseizure drugs (23.4%), antipsychotics (16.6%), anxiolytics (13.8%), and sedatives (4.1%). No significant association was found between the use of psychotropic medications and physical function. Cognitive function (β=1.489, SE 0.265, p< 0.001), comorbidities (β=-2.680, SE 1.093, p=0.014), and pain (β=-2.837, SE 0.865, p=0.001) were directly associated with physical function. Cognitive function was also indirectly associated with function through pain. The findings support prior research showing a lack of association between the use of psychotropic medications and physical function. Additional research may be needed to investigate the mediating effects of pain between cognition and physical function in older adults with dementia.

